# Gastric Stem Cell and Cellular Origin of Cancer

**DOI:** 10.3390/biomedicines6040100

**Published:** 2018-10-31

**Authors:** Masahiro Hata, Yoku Hayakawa, Kazuhiko Koike

**Affiliations:** Department of Gastroenterology, Graduate School of Medicine, the University of Tokyo, Tokyo 1138655, Japan; hatamasa1112@gmail.com (M.H.); kkoike-tky@umin.ac.jp (K.K.)

**Keywords:** gastric cancer, stem cell, gastric stem cell, stem cell niche, *Mist1*, *Lgr5*

## Abstract

Several stem cell markers within the gastrointestinal epithelium have been identified in mice. One of the best characterized is *Lgr5* (leucine-rich repeat-containing G-protein coupled receptor 5) and evidence suggests that *Lgr5*+ cells in the gut are the origin of gastrointestinal cancers. Reserve or facultative stem or progenitor cells with the ability to convert to *Lgr5*+ cells following injury have also been identified. Unlike the intestine, where *Lgr5*+ cells at the crypt base act as active stem cells, the stomach may contain unique stem cell populations, since gastric *Lgr5*+ cells seem to behave as a reserve rather than active stem cells, both in the corpus and in the antral glands. Gastrointestinal stem cells are supported by a specific microenvironment, the stem cell niche, which also promotes tumorigenesis. This review focuses on stem cell markers in the gut and their supporting niche factors. It also discusses the molecular mechanisms that regulate stem cell function and tumorigenesis.

## 1. Discovery of Gastrointestinal Stem Cells

Self-renewal and pluripotency are cardinal properties of stem cells [1]. In the gastrointestinal system, the integrity of the epithelium is maintained by long-lived stem cells. All differentiated epithelial cells are in a state of turnover and, within several days (intestines and distal stomach) or months (proximal stomach), are replaced by cells newly generated from long-lived, self-renewing stem cells. Intestinal stem cells (ISCs) in mice were identified in earlier label-retention assays, in which a nucleic acid analog was administered in combination with epithelial damage, such as irradiation [2]. The results of those assays suggest that the small intestinal crypt contains a single population of label-retaining stem cells, located just above the Paneth cells, which is solely responsible for cell replacement and is resistant to irradiation-induced injury.

In 2007, Clevers et al. identified *Lgr5* (leucine-rich repeat-containing G-protein coupled receptor 5) -expressing crypt base columnar cells (CBCs) residing between Paneth cells at the crypt base, by using *Lgr5*-CreERT and *Rosa-26* reporter mice [3]. Lineage-tracing experiments with those mice showed the longevity and multipotentiality of *Lgr5*-expressing cells in vivo, i.e., labeled *Lgr5*-expressing cells continuously self-renew and supply their progeny on the luminal side for their entire lifespan. Therefore, the authors concluded that *Lgr5*-expressing cells are long-lived ISCs. They also found *Lgr5*+ stem cells at the colonic gland base. Subsequent studies from this group demonstrated the stem cell function of *Lgr5*+ cells, by generating three-dimensional intestinal organoids in vitro from single *Lgr5*-expressing cells [4,5]. It was also shown that epidermal growth factor (EGF), Noggin, R-spondin and Wnts, some of which are produced by Paneth cells, are essential to maintaining intestinal organoids in culture and therefore are likely to play critical roles in the ISC niche [4,5]. However, other groups reported different stem cell populations, at the “+4” position above *Lgr5*+ stem cells [6]. These +4 stem cells, which express the markers *Bmi1, Hopx* and *Tert* divide and proliferate more slowly than *Lgr5*+ stem cells [7,8]. Moreover, while *Lgr5*+ stem cells are sensitive to intestinal damage such as radiation, die and disappear shortly after intestinal injury, +4 stem cells are resistant to intestinal damage and can compensate for the function of *Lgr5*+ stem cells through stem cell interconversion [9,10,11]. Therefore, +4 stem cells have been referred to as reserve stem cells, that is, they are normally quiescent but can actively contribute to epithelial regeneration following injury. Indeed, another group also reported that classical label-retaining cells (LRCs) may represent pre-Paneth precursors and can act as a reserve stem cell following epithelial injury in the short-term. On the other hand, in normal homeostatic conditions, rapidly dividing *Lgr5*+ CBCs continuously supply their daughter cells including LRCs, integrating earlier findings on LRCs and the current understanding of the ISC hierarchy [12].

**Figure 1:**
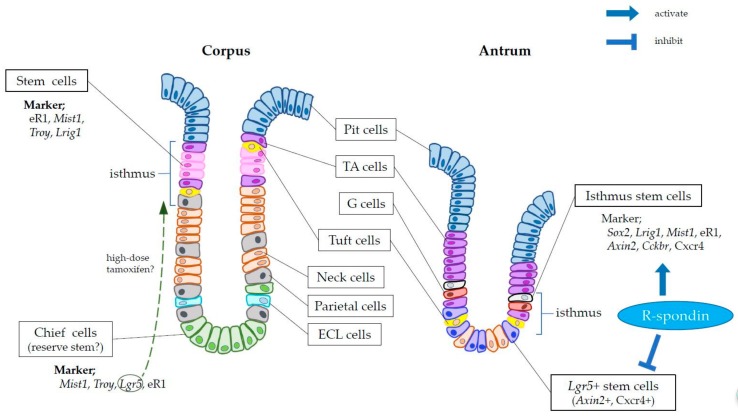
Schema of murine gastric glands and cell types. In the corpus glands (**left**), stem and transit-amplifying (TA) progenitor cells reside within the isthmus, and supply mature cell types including surface pit cells, parietal cells, neck cells, tuft cells, enterochromaffin-like (ECL) cells, and chief cells. *Mist1*, *Troy*, and Runx1 enhancer element (eR1) are expressed in both stem and chief cells, while *Lgr5* (leucine-rich repeat-containing G-protein coupled receptor 5) expression is normally restricted in chief cells. However, following high-dose tamoxifen-induced damage, aberrant *Lgr5* expression is observed within the isthmus. In the antrum (**right**), there are two distinct stem cell populations; one expresses *Lgr5* at the base, the other expresses *Sox2*, *Lrig1*, *Mist1*, eR1, or *Cckbr* (cholecystokinin B receptor) within the isthmus, and is more proliferative. *Axin2* and Cxcr4 are expressed in both populations. R-spondin activates antral isthmal stem cells but inhibits *Lgr5* expressing stem cells.

The mouse stomach is anatomically divided into two parts. Corpus glands in the proximal stomach contain cells that secrete gastric acid and digestive enzymes, while cells in the antral glands of the distal stomach secrete hormones such as gastrin and somatostatin. Both corpus and antral glands contain the isthmus region, where the glands narrow and stem cells are thought to reside, but the location of the isthmus zone differs between the proximal and distal stomach [13]. In the relatively shorter antral glands, the isthmus is located at roughly the +4 to +10 position, slightly above the gland base, whereas in the relatively taller corpus glands it is found within the upper-third of the glands. Previous electron microscopy and nucleic acid analog labeling studies demonstrated that stem and progenitor cells within the isthmus regions supply their progeny, which migrate bidirectionally toward the lumen and the basal side in the corpus and antrum [14,15]. However, after the discovery of intestinal *Lgr5*+ stem cells, gastric *Lgr5*-expressing cells were identified in the antral gland base (i.e., below the antral isthmus). These apparently self-renewing, multipotent cells supply their daughter cells upwards from the base [16]. In contrast to other parts of the gastrointestinal tract, stem cells in the corpus isthmus do not express *Lgr5*, however, other specific corpus stem cell markers were subsequently identified (Figure 1). As discussed later, it has been proposed that gastric chief cells which express *Lgr5* and reside at the corpus gland base may act as a reserve stem cell after epithelial injury [17].

## 2. Markers of Gastric Stem Cells

The corpus and antral glands have different stem cell populations. Similar to *Lgr5*+ ISCs, antral *Lgr5*+ stem cells are located at the gland base (position +1 to +3) and demonstrate a “lineage tracing” ability throughout their life span [16]. However, *Lgr5*+ stem cells in the antrum are more quiescent (proliferation rate of 10–20%) than those in the intestinal epithelium. In addition, in the antral isthmus, *Lgr5*-negative stem cells that are analogous to intestinal +4 stem cells have been identified. These +4 antral stem cells express *Cckbr* (cholecystokinin B receptor), *Sox2*, *Lrig1*, *Bhlha15* (also known as *Mist1*) and Runx1 enhancer element (eR1) and have a higher proliferation rate (~40–50%) than antral *Lgr5*+ stem cells, whereas intestinal +4 stem cells are less proliferative [18,19,20,21,22,23]. A recent report identified *Axin2* as an antral stem cell marker expressed by isthmal proliferating cells and basal *Lgr5*+ cells [24] (Figure 1). The observation that the ablation of *Lgr5*+ cells does not impair the lineage tracing ability of *Axin2*+ cells confirmed the presence of *Lgr5*-negative, *Axin2*+ stem cells in the isthmus. Furthermore, while the administration of R-spondin, a ligand for *Lgr5*, inhibits the expansion and tracing of *Lgr5*-derived clones, it promotes lineage tracing by *Axin2*+ cells. These findings suggest that R-spondin inhibits stem cell function in *Lgr5*+ cells and/or that it activates *Axin2*+ cells via other receptors, such as Lgr4. The exact stem cell hierarchy in the gastric antrum, including basal *Lgr5*+ cells and isthmal progenitors, remains to be determined.

In the corpus, detailed analyses of potential corpus stem or progenitor cell markers such as *Sox2* or *Lrig1* have been performed only recently [19,20,25], as most research attention is devoted to gastric chief cells due to their potential proliferation and dedifferentiation ability. As a mature cell type, gastric chief cells secrete several digestive enzymes. They are found at the very base of the corpus glands, not at the isthmus region.

In 2010 a study of *Mist1*-CreERT mice, which was supposed to specifically mark chief cells, suggested that chief cells may be able to transdifferentiate into metaplastic cells following injury [26]. The authors showed that metaplastic cells in the stomach exposed to protonophores (DMP-777 and L-635) or *Helicobacter* infection are traced by their *Mist1*-lineage and are presumably derived from *Mist1*+ chief cells. However, using *Lgr5*-EGFP-IRES-CreERT mice originally developed by Clevers’ group, the same group demonstrated that *Lgr5*+ chief cells fail to give rise to metaplasia in the same drugs or *Helicobacter* infection model [27].

In 2013, Clevers′ group, studying *Troy*-CreERT mice, identified a subset of chief cells that express *Troy*. The cells can proliferate and supply daughter cells upwards from the gland base at a rare rate and such ability in *Troy*+ chief cells are more evident particularly when isthmus stem/progenitor cells are injured [28]. This again raised the issue of the dedifferentiation ability of chief cells, which also coincided with the recognition of a greater-than-expected degree of cellular plasticity in the intestinal epithelium. For example, it was reported that *Dll1*+ intestinal secretory progenitors appear to interconvert to stem-like cells following epithelial injury and under rare circumstances, that even more differentiated Dclk1+ tuft cells may dedifferentiate into stem-like, cancer-initiating cells [29,30,31]. These results provided support for the potential dedifferentiation or transdifferentiation of another differentiated cell population, gastric chief cells.

Nonetheless, the work of our group and others raised doubts regarding the ability of drug-induced injury alone to induce stemness in terminally differentiated chief cells. Indeed, we showed that both *Mist1* and *Troy* are expressed not only by gastric chief cells, but also by long-lived isthmus stem cells, and that *Mist1*-derived or *Troy*-derived clones expand bidirectionally from the isthmus, rather than migrating upward from the basal chief cell zone (Figure 1). Lineage tracing of *Mist1*+ cells was not impaired by chief cell ablation in *Lgr5*-DTR mice, but was impaired by isthmus stem cell ablation using 5-fluorouracil [22]. These findings call into question the true source of the tracing events in normal or metaplastic glands. In addition, to induce gene recombination, CreERT mice must be given tamoxifen, which in itself causes severe gastric epithelial injury [32]. Moreover, in studies using knock-in mice, in which one copy of the target gene is replaced by CreERT sequences, haploinsufficiency of the target gene may affect cellular function and the in vivo phenotype [22]. Thus, the tracing or proliferation ability of chief cells may differ from that of cells in a normal state.

A subsequent study also emphasized the importance of isthmus stem cells in gastric mucosal maintenance, by using eR1-CreERT to mark actively cycling isthmus stem/progenitors (and a rare population of chief cells) in mice [21]. Paradoxically, another group recently proposed cellular plasticity and dedifferentiation of chief cells following high-dose tamoxifen-induced injury, in a study using another *Lgr5*-2A-CreERT mouse line which marks broader chief cell population than the original *Lgr5*-EGFP-IRES-CreERT mice [17]. Nevertheless, our group more recently demonstrated that, even in the original *Lgr5*-EGFP-IRES-CreERT mice, *Lgr5* gene expression and CreERT-induced gene recombination occurs in the isthmus region, which is physically distinct from the chief cell region, following a high-dose-tamoxifen pulse protocol [33]. Thus, although isthmus expression of *Lgr5*-2A-CreERT transgene after high-dose tamoxifen injury has yet to be confirmed, in our opinion, there seems to be no available mouse lines that specifically target chief cells alone, without marking isthmus progenitors and studies on chief cell dedifferentiation might therefore be vulnerable to mis- or over-interpretation.

The cardia, a minimal segment in the stomach, is situated nearest the esophagus in the gastric corpus. Cardia glands are quite similar to antral glands in many aspects, as they contain stem cells that express *Lgr5* at the base and *Cckbr* in the isthmus [16,34]. Both of these stem cell types have been implicated in the development of Barrett′s esophageal metaplasia [34,35].

## 3. Cell-of-Origin of Gastric Cancer

Cancer arises from the accumulation of multiple genetic and epigenetic alterations. Stem cells in the affected organs are most likely to be the origin cells of cancer because they should be able to self-renew and survive for a long period after multiple cell divisions [13]. In the CreERT mouse system, oncogenic mutations can be induced in specific cell types, allowing the cellular origin of cancer to be identified. Knocking out the *Apc* (adenomatous polyposis coli) gene in *Lgr5*+ ISCs rapidly generates adenomas in the mouse small intestine and colon, whereas *Apc* knockout in differentiated mature cells does not [36]. Although *Apc* gene mutation is less frequent in human gastric cancer than in colorectal cancer, knocking out the *Apc* gene in gastric antral stem cells leads to the development of adenoma or intramucosal well-differentiated carcinoma. While antral stem cells expressing *Lgr5*, *Mist1* or *Sox2* may be among the gastric cancer origin cells in the setting of *Apc* loss [16,23,37], *Lgr5*+ cells have been implicated in more invasive types of gastric cancer, characterized by the simultaneous loss of *Pten* and *Smad4*, or by the loss of *Lats1* and *Lats2* [38,39].

In studies on corpus gastric cancer, *Atp4b*-Cre mice have been frequently used. *Atp4b*-Cre mice crossed with mice that conditionally overexpress the intracellular domain of Notch develop intestinal-type dysplasia in the corpus, while the progeny of those crossed with *Cdh1*^flox/flox^ mice, in combination with the knockout of *Tp53* or mutant *Kras*, develop a diffuse-type gastric cancer [40,41]. However, this does not point to *Atp4b*-expressing parietal cells as the source of these cancers, since Cre is expressed constitutively from the fetal phase and oncogenic alterations likely change the cell fate of *Atp4b*-Cre-expressing fetal progenitors into stem-like cells. *Mist1*+ cells have been identified as a source of corpus cancers in studies using *Mist1*-CreERT mice. Like *Atp4b*-Cre mice, when the active Notch intracellular domain is expressed by cells of *Mist1*+ lineage, intestinal-type dysplasia develops both in the mouse gastric corpus and antrum [22,42]. In contrast to the antrum, loss of *Apc* alone in *Mist1* lineage does not induce dysplasia or tumor formation in the corpus. However, the simultaneous induction of mutant *Kras* and *Apc* loss results in the rapid development of intestinal-type gastric cancer even in the corpus [22].

This unique phenotype in the corpus and in the setting of *Apc* loss may be related to the pathogenesis of human gastric cancer, the so-called Correa pathway, in which gastric atrophy and intestinal metaplasia precede dysplasia and cancer. In mouse models, activation of the Kras-MAPK pathway leads to the development of metaplasia in the corpus. In fact, in *Mist1*-CreERT; LSL-*Kras*^G12D^ mice, metaplastic cells rapidly replace the entire epithelium of the gastric corpus [22,43]. The requirement for Kras activation for gastric cancer development in *Apc*-deleted corpus glands suggests that gastric metaplasia and a loss of the normal cell lineage (gastric atrophy) are necessary for cancer initiation in the corpus.

Because *Mist1* labels both chief cells and stem cells, the origin of metaplasia in *Mist1*-CreERT; LSL-*Kras*^G12D^ mice has been a matter of debate [44,45,46]. However, given that metaplastic cells arise in *Mist1*-CreERT; LSL-*Kras*^G12D^; *Lgr5*-DTR mice even after the ablation of *Lgr5*+ chief cells, most of the metaplastic cells are likely to be derived from *Mist1*+ isthmus stem cells, or at least from *Lgr5*-negative *Mist1*+ cells [22]. The induction of mutant *Kras* in the upper isthmus region of *K19*-CreERT or *Tff1*-Cre mice results in the development of a similar metaplasia and supports the notion that isthmus stem/progenitors are the predominant source of metaplasia [22,47,48]. Although the research group using *Lgr5*-2A-CreERT mice claimed that *Lgr5*+ chief cells gave rise to cancer following high-dose-tamoxifen-induced injury and mutant *Kras* expression, Kras activation alone does not cause histological cancer, but instead metaplasia, as in other Kras models. In addition, as seen in the original *Lgr5*-EGFP-IRES-CreERT mice, the high-dose administration of a reagent that causes gene recombination might also induce unexpected responses, such as aberrant expression of the target gene outside the chief cell zone [33]. There is also another argument from a group using eR1-CreERT mice that isthmus-derived Kras-activated clones and chief-cell-derived Kras-activated clones have distinct characters, with the former giving rise to foveolar hyperplasia and the latter to spasmolytic polypeptide-expressing metaplasia (SPEM) [21]. Although we have a different opinion and interpretation of their results [45]. However, all mice with mutant *Kras* eventually develop SPEM at the base of the metaplastic glands and glands in the *Kras*-mutated stomach predominantly contain aberrant MUC4+ cells with Alcian blue-positive ectopic mucins [47]. Distinguishing between SPEM and other types of metaplasia could be an interesting experimental approach, but it is unlikely to be relevant to a determination of the cellular origin of metaplasia or pathogenesis in humans [49] given that the ultimate phenotypes in all Kras models are essentially indistinguishable.

Aberrant E-cadherin protein may contribute to the tumorigenesis of diffuse-type carcinoma and signet ring cell carcinoma (SRCC). When the *Cdh1* gene was floxed out in *Mist1*+ cells, a few clusters of SRCC-like cells arose in the isthmus region of the corpus. However, these SRCC-like cells gradually disappeared within several weeks or months, because E-cadherin regulates cell adhesion and is therefore likely to be essential for cell survival. Interestingly, chronic *H. felis* infection in these mice enabled SRCC-like cells to survive and expand over time, eventually giving rise to diffuse-type cancer [22]. Thus, the chronic inflammation induced by *Helicobacter* infection may play an important role in the tumorigenesis of not only intestinal-type cancer but also SRCC. Given that the additional mutation of the *Tp53* gene causes a more invasive diffuse-type gastric cancer in the infected mice, these genetic mutations and external inflammatory stimuli may coordinately drive survival by preventing anoikis after the loss of E-cadherin expression.

## 4. Gastric Stem Cell Niche

Gastrointestinal stem cells are confined to the stem cell zone and are thus critical to the maintenance of longevity and multipotentiality. Previous works suggested that both stromal cells and a subset of epithelial cells constituted the stem cell niche, responding to the paracrine secretion of essential factors [5,50]. As described above, Wnt, R-spondin, Notch, bone morphologic protein (BMP), Noggin and EGF signals are important niche factors for ISCs [51,52,53,54]. In the intestine, R-spondin and Wnts are secreted from mesenchymal cells that include *Foxl1*+ telocytes and CD34+ pericytes, both of which maintain ISC functions and tissue homeostasis. In addition, *Grem1*+ stromal cells surrounding the crypt base may also support the ISC niche [50,51,52,53,54]. Epithelial Paneth cells express Wnts, EGF and Delta-like (DLL) family protein, and activate *Lgr5*+ ISCs through direct physiological contact [5]. *Dll1*+ progenitors at the +5 position may also activate Notch expressing ISCs [29].

A recent report suggested that in the stomach, R-spondin 3 from myofibroblasts contributes to the antral stem cell niche and predominantly activates *Lgr5*-negative *Axin2*+ stem cells (presumably expressing Lgr4) [24]. On the other hand, antral *Lgr5*+ stem cells are supported and activated by Wnt signaling proteins via at least in part a Frizzled-7 receptor [55]. In addition, we reported that gastrin from antral G-cells and acetylcholine from the Dclk1+ tuft cell may play unique roles within the antral stem cell niche. Gastrin is generated by G-cells residing near the antral isthmus region, where *Cckbr*+ stem cells (the gastrin receptor) are also found. Interestingly, a precursor form of gastrin known as progastrin, stimulates *Cckbr* (the gastrin receptor)-expressing stem cells and promotes antral carcinogenesis, while amidated gastrin does not. Since gastrin levels highly affect antral proliferation and carcinogenesis, the gastrin-dependent interaction between *Cckbr*+ stem cells and G-cells is likely to be involved in the pathogenesis of various antral diseases as a hormonal gastric stem cell niche [18,34,56]. *Lgr5*+ cells in the gastric antrum strongly express muscarinic acetylcholine receptors and are regulated by acetylcholine, released from tuft cells and stromal nerves [30]. We also identified a perivascular niche for gastric stem cells in both the corpus and antrum. Vascular endothelial cells adjacent to gastric stem cells expressed the chemokine Cxcl12 and interacted with the Cxcr4+ innate lymphoid cells, as well as Cxcr4+ antral stem cells [23]. During chronic inflammation and tumorigenesis, the Cxcl12/Cxcr4-dependent perivascular niche expands and promotes cancer development, partly through the production of Wnt5a from innate lymphoid cells [22]. Further efforts are needed for a detailed characterization of the various factors and components involved in the gastric stem cell niche and thus in gastric stem cell biology.

## 5. Conclusions and Perspective

Gastric epithelial homeostasis is maintained by long-lived stem cells surrounded by a supportive stem cell niche. Gastric cancer arises from stem cells that have accumulated gene mutations and from the subsequent aberrant expansion of the stem cell niche. Effective curative therapies for gastric cancer will require further studies aimed at identifying the key driver gene mutations in gastric cancer development and the targetable stem/cancer cell niche compartments.
